# Traumatic injury pattern is of equal relevance as injury severity for experimental (poly)trauma modeling

**DOI:** 10.1038/s41598-019-42085-1

**Published:** 2019-04-05

**Authors:** Bing Yang, Katrin Bundkirchen, Christian Krettek, Borna Relja, Claudia Neunaber

**Affiliations:** 1 Trauma Department, Hannover Medical School, Hannover, Germany; 2 Department of Trauma, Hand and Reconstructive Surgery, University Hospital Frankfurt, Goethe University, Frankfurt, Germany

## Abstract

This study aims to elaborate the relevance of trauma severity and traumatic injury pattern in different multiple and/or polytrauma models by comparing five singular trauma to two different polytrauma (PT) models with high and one multiple trauma (MT) model with low injury-severity score (ISS). The aim is to provide a baseline for reducing animal harm according to 3Rs by providing less injury as possible in polytrauma modeling. Mice were randomly assigned to 10 groups: controls (Ctrl; n = 15), Sham (n = 15); monotrauma groups: hemorrhagic shock (HS; n = 15), thoracic trauma (TxT; n = 18), osteotomy with external fixation (Fx; n = 16), bilateral soft tissue trauma (bSTT; n = 16) or laparotomy (Lap; n = 16); two PT groups: PT I (TxT + HS + Fx; ISS = 18; n = 18), PT II (TxT + HS + Fx + Lap; ISS = 22; n = 18), and a MT group (TxT + HS + bSTT + Lap, ISS = 13; n = 18). Activity and mortality were assessed. Blood gas analyses and organ damage markers were determined after 6 h. Significant mortality occurred in TxT, PT and MT (11.7%). Activity decreased significantly in TxT, HS, both polytrauma and MT *vs*. Ctrl/Sham. PT-groups and MT had significantly decreased activity *vs*. bsTT, Lap or Fx. MT had significantly lower pCO_2_ *vs*. Ctrl/Sham, Lap or bsTT. Transaminases increased significantly in PT-groups and MT *vs*. Ctrl, Sham or monotrauma. Traumatic injury pattern is of comparable relevance as injury severity for experimental multiple or (poly)trauma modeling.

## Introduction

Trauma is one of the leading causes of mortality worldwide^1^. Epidemiological studies on multiple trauma have shown that 81% of injured patients suffer from fractures of extremities, 58% from thoracic trauma, and 50–65% from traumatic brain injuries (TBI)^2^. Although certain injuries, such as severe TBI or hemorrhage, display higher mortality rates, the combination of multiple injuries aggravates the outcome even more^3^. Complex immune response, originally initiated to limit further damage and induce healing, has been determined as a key factor for complications and fatal outcome after trauma^4,5^. Additionally, metabolic and respiratory compensatory capability contribute to posttraumatic outcome^6,7^. For example, patients who initially survived trauma frequently develop inflammatory complications, such as pneumonia and/or multiple organ failure, during their later clinical course^8^. Despite improved treatment strategies both mortality and disability rates still remain high^2^. Therefore, a reliable *in vivo* model is necessary to investigate the physiological response pattern to polytrauma. The most common *in vivo* trauma models imply mainly two insults, with major focus on hemorrhagic shock in combination with blunt chest trauma or fracture^9–14^. This so-called double-hit trauma model of a thoracic injury with hemorrhage/resuscitation is often applied to combine the posttraumatic systemic and local inflammatory response and organ damage^9,10^. In turn, the combinatory model of hemorrhagic shock and fracture is mainly focused on fracture healing under traumatic conditions^11–14^.

Polytrauma has been frequently determined by Injury Severity Score (ISS) ≥ 16^15,16^. ISS is defined by the highest Abbreviated Injury Scale (AIS) severity code in each of the three most severely injured body regions^17,18^. However, this definition of polytrauma does not always reflect the physiological course after injury^19,20^ and, moreover, a high ISS may be caused by a severe single-system injury (monotrauma) as well^21^. Therefore, additional qualifying criteria have been recorded in the new “Berlin definition”. These include laparotomy, severe shock, admission into the intensive care unit, a systemic inflammatory response syndrome^21^, or combine the concept of different injury patterns with the addition of physiological responses^22^. Additionally, according the new “Berlin definition”, polytrauma is defined by two injuries that are greater or equal to 3 on the AIS and one or more additional factors such as hypotension, unconsciousness, acidosis, coagulopathy or elderly age. Thus, with regard to the definition of polytrauma by the ISS ≥ 16, experimental *in vivo* models which combine two insults do not fulfill the new polytrauma criteria. The relevance of traumatic injury pattern in development of early systemic inflammatory response was shown by Weckbach *et al*., comparing different two-hit trauma models with a novel murine experimental polytrauma model consisting of a blunt chest trauma, head injury, femur fracture and soft tissue injury^23^. Inclusion of a hemorrhagic shock by Denk *et al*. to this model increased the clinical relevance significantly^24,25^. Thus, this polytrauma model forms the basis for an accurate investigation of the early pathophysiology of polytrauma and subsequent therapeutic interventions. However, a long-term polytrauma model applicable for example to delayed complications initiated by MOF or sepsis has not been designed so far. To establish such a model, besides data describing the severity of traumatic injury, the injury pattern and outcome, monitoring animal activity and physiological characterization are of the highest relevance. In this study, we report about the importance of each monotrauma that is applied in the setting of an experimental polytrauma whereby a good baseline for choosing an appropriate model for long-term observations was given. Thereby, the animal suffering according to the reduction and refinement criteria of the 3Rs can potentially be reduced.

## Materials and Methods

### Animal care

The experiments were performed in accordance with the German Animal Welfare Legislation, approved by the local institutional animal care and research advisory committee of the Hannover Medical School and permitted by the Veterinary Institute for Animal Welfare of the Lower Saxony State Office for Consumer Protection and Food Safety, Germany (Approval No. 33.12-42502-04-13/1323).

Male C57BL/6NCrl mice aged 12 weeks were purchased from Charles River Laboratories (Sulzfeld, Germany). Animals were housed and fed under standardized conditions^13^. All mice undergoing surgery received inhalational isoflurane anesthesia (Baxter Deutschland GmbH, Unterschleißheim, Germany) and 1% prilocainhydrochlorid (Xylonest® AstraZeneca GmbH., Wedel, Germany) for local anesthesia. Before surgery, 5 mg/kg body weight carprofen (Rimadyl, Zoetis Deutschland GmbH, Berlin, Germany) and 1 mg/kg body weight butorphanol (Torbugesic, Zoetis Deutschland GmbH, Berlin, Germany) were subcutaneously applied. For postoperative analgesia, 0.8 mg/mL Novaminsulfon Lichtenstein 1500 mg (Zentiva Pharma GmbH, Frankfurt am Main, Germany) was added to the drinking water. During and after the surgery, infrared warming lamps and heating pads were used to keep animals’ body temperature stable. Body weight and activity of the mice were measured for all animals before trauma and before euthanasia.

### Group allocation

Mice were randomly assigned to one of ten groups. Table 1 shows the group distribution and interventions in each group. In short, two control groups were included. One group consisted of healthy animals without interventions (control, Ctrl), and another group underwent catheterization and ligation of the femoral artery without blood loss and reperfusion (Sham). Furthermore, five single injury (monotrauma) groups were analyzed: hemorrhagic shock (HS), thoracic trauma (TxT), osteotomy with external fixation (Fx), bilateral soft tissue trauma (bSTT) and laparotomy (Lap) as abdominal trauma. We also investigated two different polytrauma (PT) groups, which are PT I consisting of a combinatory TxT, HS and Fx (ISS = 18), and PT II consisting of TxT, HS, Fx and Lap (ISS = 22), respectively, and a multiple trauma (MT) group with an ISS below 16 (ISS = 13), consisted of TxT, HS, Lap and bSTT instead of Fx. All groups were scored referring to the ISS system^18^. Immediately upon intervention the animals were allowed to awake and had free access to water and food.

**Table 1:** Group allocation. 165 mice were subdivided into ten different groups. Total number of animals in each group and the injury severity score (ISS) are indicated. bsTT: bilateral soft tissue trauma; Ctrl: healthy animals without intervention; Fx: osteotomy and external fixation; HS: hemorrhagic shock; Lap: midline laparotomy; MT: multiple trauma (TxT + HS + Lap + bsTT); PT: polytrauma (PT I: TxT + HS + Fx and PT II: TxT + HS + Fx + Lap); Sham: surgical procedures without trauma; TxT: thoracic trauma.

Group	Treatment procedure	Total number [n]	ISS
Control	Healthy animals without intervention	15	0
Sham	catheter placement	15	0
Fx	Osteotomy + external fixation	16	9
Lap	Midline Laparotomy (2 cm)	16	4
HS	Hemorrhagic shock + reperfusion with Ringer solution	15	0
TxT	Thoracic trauma	18	9
bSTT	Bilateral soft tissue trauma (lower leg)	16	0
PT I	TxT + HS + Fx	18	18
PT II	TxT + HS + Fx + Lap	18	22
MT	TxT + HS + Lap + bSTT	18	13

The open femur fracture with external fixation and anterior muscle damage was given AIS of 3 comparable to the AIS given by others for this injury type^25,26^. A moderate bilateral thoracic trauma without major cardiac contusion was rated with AIS of 3. Others have provided an AIS of 3–4^25^ for the comparable type of injury, but in our model animals with major cardiac contusions due to their death have been excluded, we have chosen an AIS of 3. A minimal resection of two centimeters of cecum/large bowel (a large amount for a mouse) during a laparotomy would mimic an AIS of 3 awarded to patients with a laparotomy and colon resection without gross contamination in human injury^26^. However, since we have performed laparotomy with open abdomen, exposure and relocation of the cecum without its resection, the AIS of 2 was assigned. Bilateral soft tissue trauma and blood loss are not included in the AIS calculation and therefore assigned to AIS of 0. AIS scores for the three most severely injured areas were calculated as the sum of squares^17^.

### Induction of thoracic trauma (TxT)

Blunt thoracic trauma was induced as previously described^12^. In short, mice were fixed in a supine position after induction of anesthesia. Thoracic trauma was induced by dropping a 300 g aluminum weight from 50 cm height through a vertical tube onto a platform resting on the chest of the mice. The impact energy of the falling weight was 1.47 Joule. After dropping, mice were observed until they awoke or maintaining dynamic breathing (within 1 minute), if not, mice were immediately sacrificed. The cause of death was determined immediately after death by dissection.

### Induction of bilateral soft tissue trauma (bSTT)

Bilateral soft tissue trauma was modified from previous study^24,27^, and induced with the same device which was used for thoracic trauma. For bSTT, a 40 g aluminum weight falls from 120 cm height through a vertical tube onto a platform resting on the medial leg of the mice. The impact energy of the falling weight was 0.47 Joules. No fractures were observed.

### Induction of hemorrhagic shock (HS)

Hemorrhagic shock was induced as described previously^13^. Briefly, the femoral artery was cannulated with polyethylene tubing (Becton Dickinson and Company, Sparks, MD, USA). Blood pressure was measured with a measuring cell (FMI TBD-1222, Föhr Medical Instruments GmbH, Seeheim, Germany) and a measuring amplifier (MIO-0501 DC, Föhr Medical Instruments GmbH). Animals were bled from a physiologic mean arterial blood pressure^28^ *via* the catheter to a mean arterial blood pressure of 35 ± 5 mm Hg. Blood pressure was monitored constantly during bleeding and maintained for 90 minutes. After hemorrhagic shock, animals were resuscitated *via* the tubing with four times the shed blood volume with Ringer’s solution preheated to body temperature (37.5°C) over 30 minutes (Berlin-Chemie AG, Berlin, Germany). Afterwards, the catheter was removed, the vessels were occluded^29^ and the incision was closed with interrupted sutures.

### Induction of osteotomy and attachment of external fixation (Fx)

A longitudinal approach was performed and the skin, as well as the *tensor fasciae latae* was opened. Afterwards, the *vastus lateralis biceps femoris* muscles were split bluntly, and the full length of the femur was exposed. A standardized external fixator system (MouseExFix simple L 100%, RISystem AG, Davos, Switzerland) was attached to the femur to stabilize the following osteotomy^13^. Osteotomy at the middle of the femur was induced by a 0.44 mm Gigli wire saw (RISystem AG). Afterwards the *fascia lata* was closed with continuous and the skin with interrupted sutures.

### Induction of laparotomy (Lap)

In a supine position a two cm midline laparotomy was carefully performed. One mL 0.9% sodium chloride solution, preheated to body temperature (37.5°C), was administered to the cavity in order to compensate fluid loss due to opening of the abdomen cavity. Afterwards, the abdominal muscle was closed with a continuous suture and the skin with interrupted sutures, respectively.

### Activity score

For quantification of the activity, a previously described activity scoring system was used^30^. It differentiates the spontaneous activity, the response to exogenous stimuli, and the amount of spontaneous food intake. The score diverges from one to six, with six being very active, and one being moribund (see Table 2). Activity of mice was measured before trauma induction and before sacrifice. Activity loss was determined by subtracting the activity value which was obtained before sacrifice from the value that was determined before surgery.

**Table 2:** Activity Score. Activity was defined from very active (score 6) to moribund (score 1) according to the activity scoring system.

Level	Quality	Characteristics of behavior
6	Very active	Strong, curious, fast motions
5	Active	Curious, fast, sporadic, activity breaks
4	Reduced activity	Attentive, frequent activity breaks
3	Quiet	Disinterested on environment, rare activity, sleepy
2	Lethargic	No activity, persist in one position, no food uptake
1	Moribund	No activity, reduced vital functions, death is expected

### Blood sampling for determination of organ damage markers

Animals were sacrificed under deep anesthesia with isoflurane six hours after trauma induction. Heparinized blood was obtained *via* cardiac puncture. 100 μL were used for blood gas analysis, and the remaining blood was centrifuged at 2500 × g for five minutes at room temperature (Eppendorf 3200, Hamburg, Germany). Plasma was stored at −80°C for later analysis of alanine aminotransferase (ALT), aspartate aminotransferase (AST), blood urea nitrogen (BUN), lactate dehydrogenase (LDH) and creatine phosphokinase (CPK) using Spotchem EZ SP-4430 device (Arkray global business Inc., Kyoto, Japan).

### Blood gas analyses

During the complete experimentation period blood pressure and temperature were continuously monitored. Arterial blood gas analyses (BGAs) were performed directly after sacrifice. pH value, carbon dioxide partial pressure (pCO_2_ in mm Hg), partial pressure of oxygen (pO_2_ in mm Hg), oxygen saturation (sO_2_ in %), hydrogen carbonate (HCO_3_^−^ in mmol/L) and base excess (BE) were measured by BGA analyzer (ABL 825, Radiometer, Copenhagen, Denmark).

### Statistical analysis

Statistical analysis was performed using GraphPad Prism 6 (GraphPad Software, Inc., San Diego, CA). All data were nonparametric (analyzed by histogram and Shapiro-Wilk test). Comparison among groups was performed using the Kruskal-Wallis test with Dunn’s correction for multiple comparison. Chi-square test was applied for the analyses of survival. Here, each group has been compared to another group. Results were expressed as median and interquartile range unless stated otherwise. Probability value less of <0.05 was considered statistically significant.

## Results

### Survival

**Figure 1:**
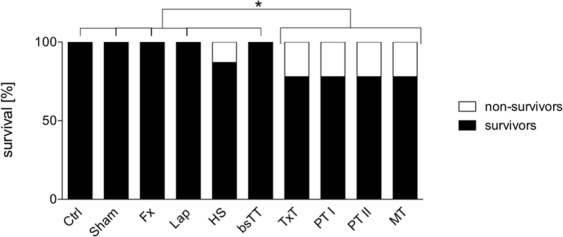
Survival analysis. Total survival during experimentation is shown. bsTT: bilateral soft tissue trauma; Ctrl: healthy animals without intervention; Fx: osteotomy and external fixation; HS: hemorrhagic shock; Lap: midline laparotomy; MT: multiple trauma (TxT + HS + Lap + bsTT); PT: polytrauma (PT I: TxT + HS + Fx and PT II: TxT + HS + Fx + Lap); Sham: surgical procedures without trauma; TxT: thoracic trauma. *p < 0.05 *vs*. indicated.

In total, 165 animals were used for this study, of which 19 mice died (overall mortality of 11.7%). In animals with TxT a mortality rate of 22% was observed. Post-mortem examination showed that 16 mice died immediately after the thoracic trauma procedure (88.9%) due to cardiac rupture, pericardial laceration, laceration to a coronary artery or severe lung contusion; two animals died during the induction of hemorrhagic shock, one mouse died for unknown reasons. No mortality was observed among the following groups: Ctrl, Sham, Fx, Lap and BsTT (see Table 3). Significant mortality was observed in TxT, PT I, PT II and MT compared to Ctrl, Sham, Fx, Lap and bsTT, respectively (p < 0.05, Table 3, Fig. 1). A trend to increased mortality in the HS group *vs*. Ctrl or Sham (p = 0.0716), *vs*. Fx or Lap (p = 0.0655) and *vs*. bsTT (p = 0.0859) was observed (Fig. 1).

**Table 3:** Mortality. The mortality rates among all groups are indicated. bsTT: bilateral soft tissue trauma; Ctrl: healthy animals without intervention; Fx: osteotomy and external fixation; HS: hemorrhagic shock; Lap: midline laparotomy; MT: multiple trauma (TxT + HS + Lap + bsTT); PT: polytrauma (PT I: TxT + HS + Fx and PT II: TxT + HS + Fx + Lap); Sham: surgical procedures without trauma; TxT: thoracic trauma.

Group	Total size (n)	Mortality (n)	Mortality (%)
Control	15	0	0
Sham	15	0	0
Fx	16	0	0
Lap	16	0	0
HS	15	2	13
TxT	18	4	22
bSTT	16	0	0
PT I	18	4	22
PT II	18	4	22
MT	18	4	22

### Activity

**Figure 2:**
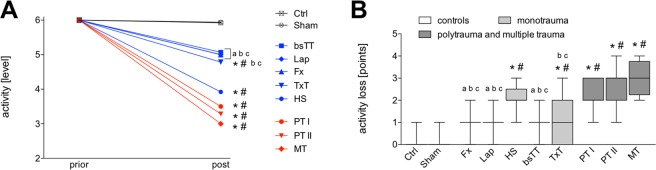
Activity score. Panel (A) shows the activity levels prior and six hours after experimentation (post). Panel (B) shows the activity loss. bsTT: bilateral soft tissue trauma; Ctrl: healthy animals without intervention; Fx: osteotomy and external fixation; HS: hemorrhagic shock; Lap: midline laparotomy; MT: multiple trauma (TxT + HS + Lap + bsTT); PT: polytrauma (PT I: TxT + HS + Fx and PT II: TxT + HS + Fx + Lap); Sham: surgical procedures without trauma; TxT: thoracic trauma. p < 0.05 *vs*. indicated or * *vs*. Ctrl; # *vs*. Sham; a *vs*. PT I; b *vs*. PT II; c *vs*. MT.

Activity behavior in Ctrl and Sham before sacrifice remained at the highest level compared to the animals activity prior experimentation (Fig. 2). Activity behavior in HS and TxT groups as well as in both polytrauma groups and in the MT group was significantly decreased before sacrifice compared to the animals activity level before experimentation, or compared to activity levels in Ctrl and Sham groups prior sacrifice, respectively (p < 0.05, Fig. 2). Prior sacrifice, activity levels of PT I, PT II and MT groups were significantly decreased compared to Fx, Lap or bsTT, respectively (p < 0.05, Fig. 2). PT II and MT group had significantly decreased activity levels compared to the TxT group (p < 0.05, Fig. 2).

### Blood gas analysis

**Figure 3:**
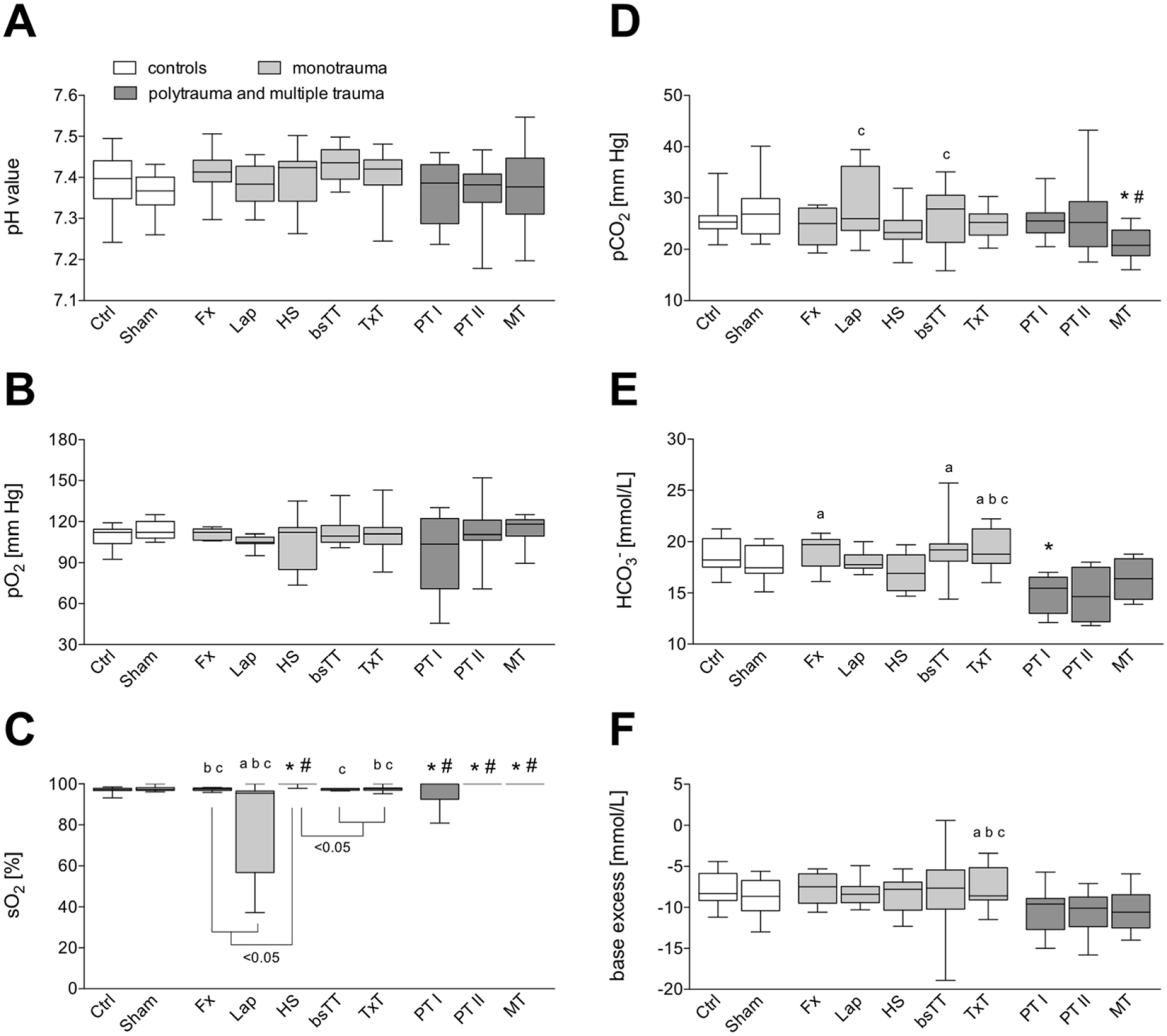
Blood gas analysis. Panel (A) shows pH values of all groups six hours after experimentation. pO_2_ (**B**), sO_2_ (**C**), pCO_2_ (**D**), HCO_3_^−^(**E**), and base excess (**F**) values of all groups after experimentation period are shown. bsTT: bilateral soft tissue trauma; Ctrl: healthy animals without intervention; Fx: osteotomy and external fixation; HS: hemorrhagic shock; Lap: midline laparotomy; MT: multiple trauma (TxT + HS + Lap + bsTT); PT: polytrauma (PT I: TxT + HS + Fx and PT II: TxT + HS + Fx + Lap); Sham: surgical procedures without trauma; TxT: thoracic trauma. p < 0.05 *vs*. indicated or * *vs*. Ctrl; # *vs*. Sham; a *vs*. PT I; b *vs*. PT II; c *vs*. MT.

At six hours after trauma, blood gas analysis showed no significant differences between the groups concerning the pH value and pO_2_ (Fig. 3A,B).

sO_2_ value was significantly higher in the HS group compared to each other monotrauma group (Lap, Fx, bsTT, and TxT) or to Ctrl and Sham, respectively (p < 0.05, Fig. 3C). sO_2_ was significantly enhanced in both PT groups and the MT group compared to Ctrl, Sham, or Lap, respectively (p < 0.05; Fig. 3C). PT II and MT had significantly increased sO_2_ *vs*. Fx and TxT (p < 0.05, Fig. 3C). MT had significantly increased sO_2_ *vs*. each monotrauma except the HS group (p < 0.05, Fig. 3C).

pCO_2_ was significantly lower in MT group compared to Ctrl, Sham, Lap and bsTT, respectively (p < 0.05, Fig. 3D).

HCO_3_^−^ was significantly lower in PT I group compared to Ctrl, Fx, bSTT and TxT group, respectively (p < 0.05; Fig. 3E). HCO_3_^−^ was significantly higher in the TxT group compared to both PT groups and to the MT group (p < 0.05; Fig. 3E).

Base excess was significantly lower in both PT groups and MT group compared to the TxT group (p < 0.05, Fig. 3F).

**Figure 4:**
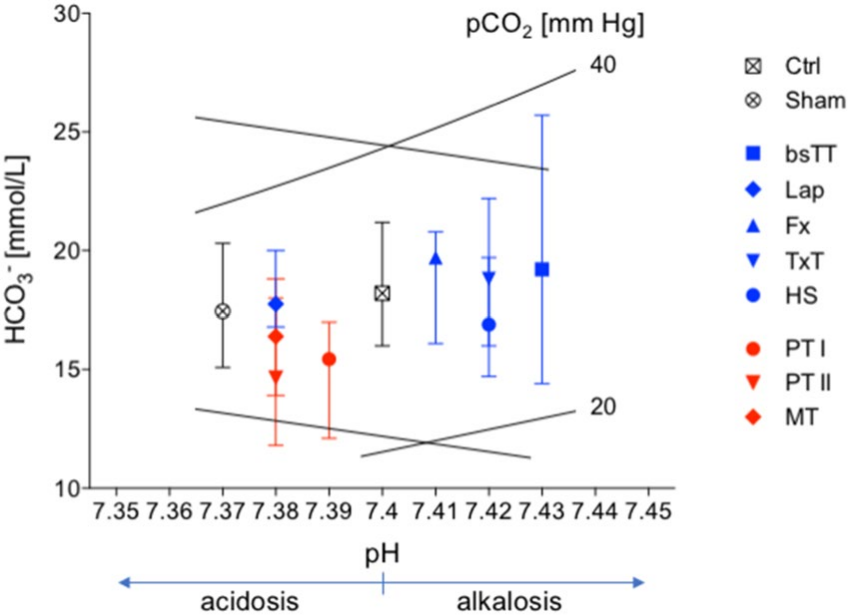
Davenport diagram showing the degree of respiratory and metabolic acidosis. The values after experimentation are shown. bsTT: bilateral soft tissue trauma; Ctrl: healthy animals without intervention; Fx: osteotomy and external fixation; HS: hemorrhagic shock; Lap: midline laparotomy; MT: multiple trauma (TxT + HS + Lap + bsTT); PT: polytrauma (PT I: TxT + HS + Fx and PT II: TxT + HS + Fx + Lap); Sham: surgical procedures without trauma; TxT: thoracic trauma.

The development of respiratory and/or metabolic acidosis is summarized in Fig. 4 as shown in the Davenport diagram.

### Organ damage markers

**Figure 5:**
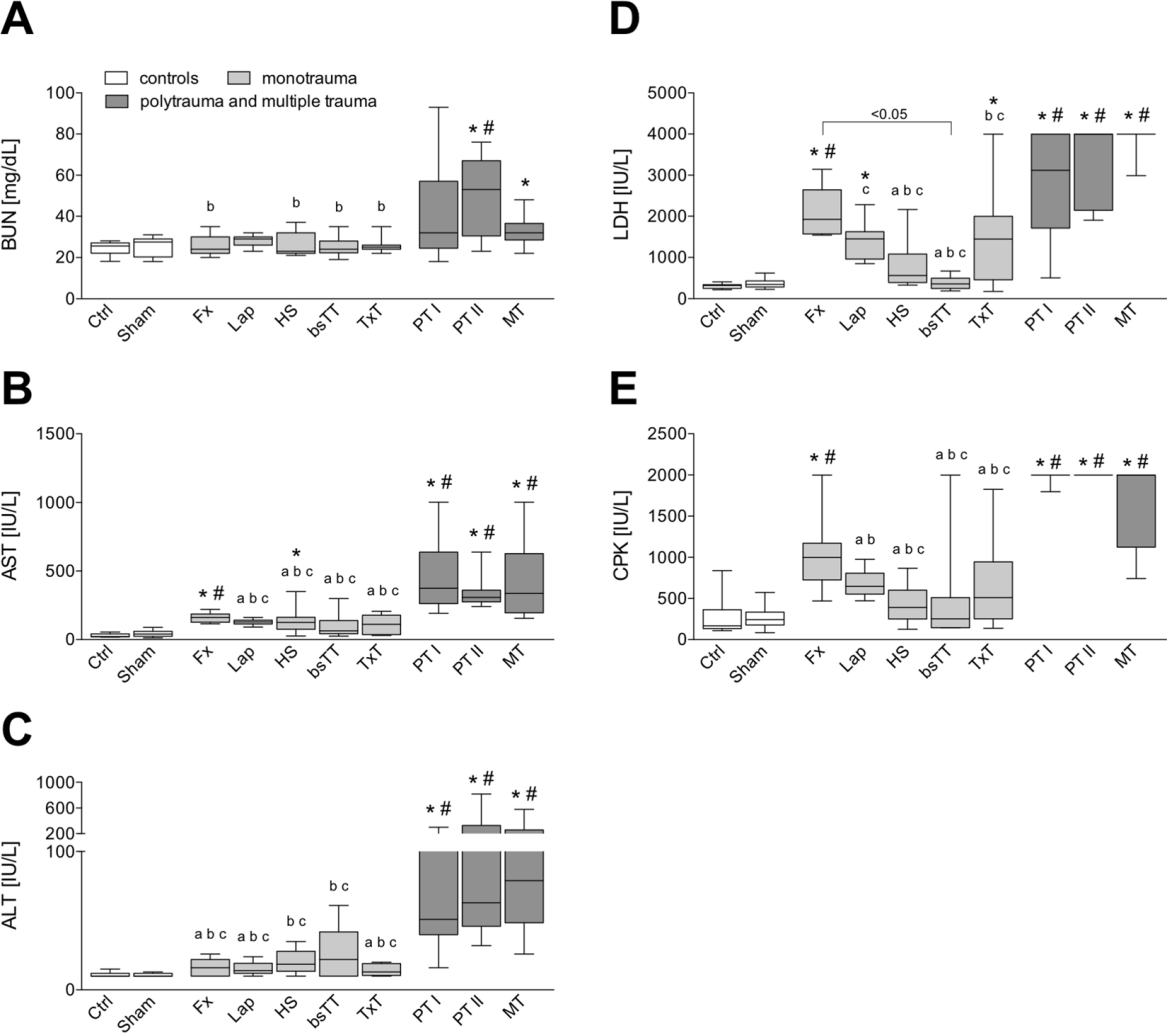
Analyses of end organ damage markers is shown. Blood urea nitrogen (BUN, **A**), aspartate aminotransferase (AST, **B**), alanine aminotransferase (ALT, **C**), lactate dehydrogenase (LDH, **D**) and creatine phosphokinase (CPK, **E**) values of all groups after experimentation period are shown. bsTT: bilateral soft tissue trauma; Ctrl: healthy animals without intervention; Fx: osteotomy and external fixation; HS: hemorrhagic shock; Lap: midline laparotomy; MT: multiple trauma (TxT + HS + Lap + bsTT); PT: polytrauma (PT I: TxT + HS + Fx and PT II: TxT + HS + Fx + Lap); Sham: surgical procedures without trauma; TxT: thoracic trauma. p < 0.05 *vs*. indicated or * *vs*. Ctrl; # *vs*. Sham; a *vs*. PT I; b *vs*. PT II; c *vs*. MT.

PT II group had a significantly higher concentration of BUN compared to Ctrl, Sham, Fx, HS, bSTT and to the TxT group (p < 0.05, Fig. 5A). BUN was significantly increased in MT compared to Ctrl (p < 0.05, Fig. 5A).

Compared to Ctrl, AST was significantly increased in Fx, HS, both PT as well as in the MT group (p < 0.05, Fig. 5B). Compared to Sham, AST was significantly increased in Fx, both PT and in the MT group (p < 0.05, Fig. 5B). Both PT groups and the MT group had significantly enhanced AST levels compared to each monotrauma except the Fx group (p < 0.05, Fig. 5B).

ALT increased significantly in each PT group and in the MT group compared to Ctrl and Sham, respectively (p < 0.05, Fig. 5C). Significantly higher concentration of ALT in the PT I group compared to montrauma groups Fx, Lap and TxT was observed (p < 0.05, Fig. 5C). In PT II and MT, ALT levels were significantly increased compared to each monotrauma group (p < 0.05, Fig. 5C).

Compared to Ctrl, LDH was significantly increased in Fx, Lap, TxT, both PT and in the MT group (p < 0.05, Fig. 5D). Compared to Sham, LDH was significantly increased in Fx, PT I, PT II and in the MT group (p < 0.05, Fig. 5D). The Fx group had significantly increased LDH level compared to both controls and bsTT (p < 0.05, Fig. 5D), but there were no significant changes compared to PT or MT groups. LDH concentration in PT I group was significantly higher compared to HS and bSTT (p < 0.05, Fig. 5D). LDH in the PT II was significantly increased compared to HS, bSTT and TxT, respectively (p < 0.05, Fig. 2D). MT had significantly higher LDH *vs*. Lap, HS, bSTT and TxT (p < 0.05, Fig. 5D). PT II and MT groups exerted highest LDH values, which reached the upper assay detection limit. Therefore, the concentration in those two groups may be higher in real as indicated, and this remains to be elaborated in further studies.

CPK increased significantly in Fx, both PT and in the MT group compared to either Ctrl or Sham (p < 0.05, Fig. 5E). In PT I and PT II groups CPK increased significantly compared to each monotrauma group except the Fx group (p < 0.05, Fig. 5E). CPK concentration in the MT group was significantly increased compared to monotrauma groups HS, bSTT, and TxT group (p < 0.05, Fig. 5E). In both PT groups as well as in the MT group CPK values reached the upper assay detection limit. Therefore, the concentration in those groups may be higher in real as indicated, and this remains to be elaborated in further studies.

## Discussion

Five singular trauma models were compared with two different polytrauma (PT) models with high ISS and a multiple trauma model with low ISS, in order to elaborate the relevance of trauma severity and traumatic injury pattern in PT modeling, and to provide a baseline for reducing the animal harm according to 3Rs. Only a few standardized and reproducible PT models exist, which mainly address the early inflammatory response after PT and provide a good basis for further investigation of the early pathophysiology of PT and the evaluation of early therapeutic interventions^23–26,31^. Yet, the clinical situation with delayed mortality caused by infectious complications highlights the importance of a long-term PT model. Therefore, data that describe the traumatic injury severity, its pattern but also animal activity or experimental outcomes and physiologic characteristics are of high relevance. Our study provides insights into the relevance of singular trauma that is applied in an experimental PT, and moreover, may provide a good baseline for choosing the appropriate model for long-term observations, since specifically the animal harm according to the reduction and refinement criteria of the 3Rs may be reduced. The data show, that animals from both PT models and from the multiple trauma model survived for at least 6 h with a mortality rate of 11.7%. The observed mortality was caused by thoracic trauma (22% in groups with TxT) or HS (13% in isolated HS group). Similarly, decreased activity was observed among the same groups. Furthermore, a trend to a metabolic acidosis with a decrease in bicarbonate and a negative base excess, which mimics the clinical situation in severely injured patients who develop complications in their later clinical course, have been observed among the PT groups and the multiple trauma group. While some monotrauma groups, as expected, exerted increased levels of transaminase, among both PT groups and the multiple trauma group a prominent organ damage to liver and musculature after 6 h occured. Data are summarized in Table 4.

**Table 4:** Summary of the study results. bsTT: bilateral soft tissue trauma; Ctrl: healthy animals without intervention; Fx: osteotomy and external fixation; HS: hemorrhagic shock; Lap: midline laparotomy; MT: multiple trauma (TxT + HS + Lap + bsTT); PT: polytrauma (PT I: TxT + HS + Fx and PT II: TxT + HS + Fx + Lap); Sham: surgical procedures without trauma; TxT: thoracic trauma. p < 0.05 * *vs*. Ctrl; # *vs*. Sham; a *vs*. PT I; b *vs*. PT II; c *vs*. MT.

Group	Size	Mortality	Activity	sO_2_	pCO_2_	HCO_3_^−^	Base excess	BUN	AST	ALT	LDH	CPK
	n	%		mm Hg	mm Hg	mmol/L	mmol/L	mg/dL	IU/L	IU/L	IU/L	IU/L
Ctrl	15	0	—	—	—	—	—	—	—	—	—	—
Sham	15	0	—	—	—	—	—	—	—	—	—	—
Fx	16	0	a b c	b c	—	a	—	b	* #	a b c	* #	* #
Lap	16	0	a b c	a b c	c	—	—	—	a b c	a b c	c *	a b
HS	15	13	* #	* #	—	—	—	b	a b c *	b c	a b c	a b c
TxT	15	22	b c * #	b c	—	a b c	a b c	b	a b c	a b c	b c *	a b c
bSTT	16	0	a b c	c	c	a	—	b	a b c	b c	a b c	a b c
PT I	18	22	* #	* #	—	*	—	—	* #	* #	* #	* #
PT II	18	22	* #	* #	—	—	—	* #	* #	* #	* #	* #
MT	18	22	* #	* #	* #	—	—	*	* #	* #	* #	* #

With regard to injury severity, comparable to the ISS, the type of traumatic injury pattern is of high relevance for experimental multiple or (poly)trauma modelling. Our data underline the currently ongoing discussion on the clinically used term “polytrauma” according to the ISS ≥ 16^15,16^ that does not necessarily reflect the physiological course after injury^19,20^. Though increased ISS is a risk factor for MOF^32^, it may be caused by a severe MT as well^21^. In our multiple trauma model, the ISS remained at a level of 13, but it also has confirmed the clinical situation that defines “polytrauma”. Thus, it may be suitable to implement the new “Berlin definition”^22^ in the definition of experimental PT. Most severely injured patients with poor outcomes suffer from a “multiple trauma” consisting of injuries to different systems and compartments. The most common *in vivo* models imply mainly two insults, with the focus on HS, which is frequently combined with blunt chest trauma or fracture^9–13,24,33–35^. Also combining HS with TBI is commonly applied^24,36–38^. Since fractures are frequently observed in the clinical scenario, implying these as an important impact in a trauma model is reasonable^13,38,39^. Similarly, laparotomy and/or soft tissue injury is relevant, since Chaudry *et al*. have confirmed that mortality after additional laparotomy to HS was increased^40^. Pretorius *et al*. have shown that for the induction of lung damage in HS, a combination of fracture and soft tissue injury was needed in their trauma model in baboons^41^.

However, the described experimental *in vivo* models barely meet the definition of PT with regard to the ISS ≥ 16^22^. Based on the human situation, an *in vivo* model should include three or more traumatic injury patterns, imply life threatening injuries, such as brain, chest or abdomen injury, and exert an ISS > 15. Thus, a non-lethal murine multiple trauma model with an ISS of 18 consisting of HS, femur fracture with muscle injury, and laparotomy with cecectomy was reported to better mimic the human inflammatory response after PT than the traditional murine double-hit models^26,31^. While Weckbach *et al*. compared their experimental PT model consisting of a blunt chest trauma, head injury, femur fracture and soft tissue injury with different two-hit trauma models, and on the one hand has demonstrated that injury pattern does matter^23^, importantly, others underlined the not deniable relevance of HS after experimental PT on the other hand^24,25,42,43^. End organ damage in both murine PT and HS model demonstrate elevated transaminases after two or 24 h^31^. Similar increase was observed in our monotrauma model of HS and in each experimental PT or the multiple trauma group with HS. Together with above discussed findings and with regard to our data, it appears reasonable to include HS in experimental PT modeling. However, HS which is observed in multiply traumatized patients would be assigned as ISS score of zero. Thus, the question about the relevance of the injury pattern and the ISS for experimental PT modeling emerges. However, all other injury types from the underlying study including fracture, abdominal injury and soft tissue trauma are common injuries in polytraumatized patients. Experimental PT model should imply as little injuries as possible to reduce the harm of animals according to the criteria of the 3Rs, and thus may provide a good baseline for choosing the appropriate model for long-term modeling. Researchers who focus on this field argue if increasing injury is required to reflect the inflammatory response emerging after multiple trauma^44–49^. Since we observe comparable data between PT groups, which have an ISS above 16 and the multiply traumatized group with an ISS below 13, this issue remains to be further discussed.

In our previous findings, a CT scan of TxT group has shown intrathoracic bleeding and hemopneumothorax without rib fractures, while the histological analyses reflected hemothorax and lung contusion in this model^12^. Here, cardiac rupture, pericardial laceration, laceration to a coronary artery followed by hemothorax or complex lung contusion caused the mortality after chest trauma. In each group with chest trauma, the mortality rate was 22%. There are other models of chest trauma e.g. providing a blast wave to the thorax^33,50^. The microscopic evaluation of lung samples revealed severe intraalveolar, intrabronchial, and subpleural hemorrhage as well as interstitial edema and atelectasis^33^. The model resulted in an early mortality rate of 10%. From a pathological point of view, both models successfully induce lung injury. Approximately 20% of trauma patients sustain cardiothoracic injuries^51^. Up to 15% of those require life-saving emergency surgery^52,53^. Therefore, the observed mortality appears reasonable. Interestingly, due to complications during thorax trauma, but also during anesthesia and because of the occurrence of complex fractures, Claes *et al*. report relevant mortality rates around 30%^54^. Unfortunately, the exclusive relevance of thoracic trauma was not evident. In our model, the post-injury treatment strategy did not include any potential life-saving surgeries, and the clinical situation regarding the post-injury treatment strategy was rather limited. Yet, this is a critical issue in all currently available polytrauma models. Of course, since ECMO therapy is increasingly applied for the treatment of patients with trauma^55^, its *in vivo* use is indicated for better improvement of the translational interpretation. However, since there are common complications of ECMO limiting its use in patients with trauma^55^, this strategy has to be considered carefully. Furthermore, ECMO often triggers systemic inflammation, which should be considered if the inflammatory response to polytrauma will be analyzed^56^. For patients requiring intubation, airway pressure release ventilation is an excellent mode to decrease the risk of acute lung injury^57^. On the other hand, in the present study, the observational time period was very narrow.

The activity decrease in both PT groups and in the multiple trauma group confirms previous data, showing that mice with multiple injuries had a significantly lower activity score *vs*. those with one injury^30^. A potentially reduced perfusion of the lower extremities through vessel ligation as a cause for the activity decrease can be excluded since Sham animals received vessel ligation, but showed no activity loss. In MT groups reduced activity was observed after HS and TxT, probably caused by blood loss and restricted lung function. Furthermore, the combination of TxT and HS with other injuries led to the highest activity loss, which was independent of the ISS. Interestingly, even after 6 h, a trend to metabolic acidosis has been observed among PT groups and the multiple trauma group. However, respiratory and metabolic compensatory abilities remain to be elucidated in further studies.

Among several available femur fracture models, here, external fixation model was chosen. Fracture can be fixed with intramedullary Kirschner wires or syringe needles as well, imitating human intramedullary nailing without applying locking screws^12,58^. However, single intramedullary Kirschner wires or syringe cannot provide rotational and axial stability. In our model, the fracture has been stabilized by an external fixator, thus both rotational and axial stability were given. With regard to the damage control surgery, this approach is popular^59^. Due to the short observational period in this study, we did not find any signs of infection. We are aware that also for the late mortality after trauma, TBI accounts for 46.7% of deaths, followed by pneumonia/respiratory insufficiency (23.9%) and MOF (11.7%); but here it should be considered that the main reasons for death at a later post-traumatic stage associated with TBI is often caused by non-survivable or severe CNS injury^8^. Additionally, different groups have already shown that fracture healing was significantly accelerated after TBI^60,61^. Although additional TBI will further increase the relevance of the underlying PT model, and it would likely lead to a superior model that better represents human PT with TBI, such a model is available and we have decided to study experimental PT without TBI.

The examination of end organ damage has shown a marked injury in both PT groups and in the multiple trauma group. As expected an increase in corresponding organ damage markers has been observed among monotrauma groups. AST increased in the HS model and in each experimental PT and the multiple trauma group, that included HS as well. The data demonstrate ongoing liver damage upon HS as shown before^31,62^. CPK and LDH elevated in groups with muscle injury, but those enzymes also exist in cardiomyocytes and hepatocytes, and indicate general cell damage^63,64^. Summarized, among both PT groups as well as in the multiple trauma group there was apparently a prominent general cell damage, and organ damage to liver and musculature. Interestingly, PT groups and the multiple trauma group showed marked differences and increased tissue damage *vs*. isolated trauma groups, however, no significant differences among PT groups and monotrauma group were observed. Comparison of PT I *vs*. PT II showed that laparotomy as additional insult did not significantly affect the results. Interestingly, in the MT group Fx has been replaced with bSTT, and this again neither affected the results. Therefore, the importance of each monotrauma in an experimental PT has to be thoroughly recapitulated, since the data demonstrates that the type of traumatic injury pattern is of comparable relevance as the injury severity for an adequate experimental multiple or (poly)trauma model.

### Limitations

During the short observation period of 6 h after trauma, the outcome regarding organ complications and prolonged survival were not evaluated. Furthermore, this time window is very wide for analyzing the metabolic and respiratory compensation upon trauma, and this should be performed immediately after trauma. On a technical level, all traumatic insults were induced within 2.5 h; however, in reality severe injuries occur simultaneously. Additionally, each monotrauma model has its advantages as well as disadvantages compared to other strategies to induce the same type of trauma. The significant increase of BUN in PT II groups may be caused by the application of NaCl into the abdominal cavity to compensate fluid loss after laparotomy. Additionally, sometimes assay detection limits were reached, and histological organ damage should be elaborated in further studies. In addition, the clinical situation was not fully represented, since after thoracic trauma no extensive surgery or blood transfusions etc. were applied. ISS calculation in murine models has to be performed carefully. The ISS has been used only few times in such models^25,26^, and the CT imaging was used only after TxT to visualize the injuries. Another limitation of the study is that TBI was not included, that may certainly lead to a superior model better representing human trauma with TBI. Yet, this issue needs to be carefully considered when choosing the appropriate experimental PT model, since with 51.6% TBI is still the main cause of death in trauma but half of all polytraumatized patients does not suffer from TBI^65,66^. Furthermore, we have investigated a small animal model. Although the mouse genome only matches approximately 80% of the human genome, specific advantages lead to common use of diverse mouse strains in experimental trauma studies^67^. Interestingly, large animal models are gaining more and more interest in the context of PT^68–70^. Because porcine hemodynamic responses are comparable to those in humans, such trauma models have been established^71,72^. Although pigs are easier to handle during surgical procedures, far more technical equipment and increased financial support are required^73^. Additionally, large animal models are mostly limited to physiological and mechanistic investigations since cell- and/or mediator-specific molecular probes and reagents are barely available.

## Conclusion

Since comparable results between the two different PT models with high ISS and the multiple trauma model with low ISS were obtained, this study demonstrates that the type of traumatic injury pattern and not necessarily the trauma severity is of high relevance for the experimental polytrauma modeling.
